# A Novel Antipathogenic Agent for Nonwoven Fabric

**DOI:** 10.1007/s44174-022-00001-8

**Published:** 2022-07-20

**Authors:** Sydney Simpson, Chelsey McMinn, Sherry M. Van Mondfrans, Jackson Hendry, Sean Ronayne, Stephen Dewhurst, Changyong Feng, B. Sonny Bal, Ryan M. Bock, Bryan J. McEntire

**Affiliations:** 1 QuidelOrtho Corporation, 100 Indigo Creek Dr., Rochester, NY 14620 USA; 2 SINTX Technologies, Inc., Salt Lake City, UT 84119 USA; 3 Department of Microbiology and Immunology, School of Medicine and Dentistry, University of Rochester, Rochester, NY 14642 USA; 4 Department of Biostatistics and Computational Biology, School of Medicine and Dentistry, University of Rochester, Rochester, NY 14642 USA

**Keywords:** Silicon nitride, Antiviral, Nonwoven fabric, Pad-dry-cure, SARS-CoV-2

## Abstract

Medical-grade masks and N95 respirators containing non-woven fibers are designed to prevent the spread of airborne diseases. While they effectively trap respiratory droplets and aerosols, they cannot lyse entrapped pathogens. Embedded antimicrobial agents such as silver, copper, zinc, iodine, peptides, quaternary ammonium salts, or nanoparticles have been used to overcome this limitation. However, their effectiveness remains debatable because these materials can be toxins, allergens, irritants, and environmental hazards. Recently, silicon nitride (Si_3_N_4_) was found to be a potent antipathogenic compound, and it may be an ideal agent for masks. In powder or solid form, it is highly effective in inactivating bacteria, fungi, and viruses while leaving mammalian tissue unaffected. The purpose of this study was to serially assess the antiviral efficacy of Si_3_N_4_ against SARS-CoV-2 using powders, solids, and embedded nonwoven fabrics. Si_3_N_4_ powders and solids were prepared using conventional ceramic processing. The “pad-dry-cure” method was used to embed Si_3_N_4_ particles into polypropylene fibers. Fabric testing was subsequently conducted using industrial standards—ISO 18184 for antiviral effectiveness, ASTM F2299 and EN 13274-7 for filtration efficiency, EN 14683 for differential pressure drop, and ISO 18562-2 for particle shedding. A modification of ISO 18562-3 was also employed to detect ammonia release from the fabric. Antiviral effectiveness for Si_3_N_4_ powders, solids, and embedded fabrics were 99.99% at ≤ 5 min, ~ 93% in 24 h, and 87% to 92% in 120 min, respectively. Results of the standard mask tests were generally within prescribed safety limits. Further process optimization may lead to commercial Si_3_N_4_-based masks that not only “catch” but also “kill” pathogenic microbes.

## Introduction

Textiles have been an essential part of human history. Traditional woven fabric is made from natural yarns (wool, cotton, silk, coir, hemp, linen, hair, etc.); but today, synthetic or blended fibers (polyester, acrylic, nylon, rayon, latex, etc*.*) are ubiquitous in everyday life [1]. Nonwoven fabric (mostly polyethylene, polypropylene, and polyester or cellulosic fibers) was developed in the latter half of the twentieth century and has supplemented or replaced many yarn-based textiles. Nonwovens have found product applications as apparel, elastomers, wipes, absorbents, and filters [2]. Of particular importance is their use for medicinal purposes, including gowns, drapes, covers, caps, wound dressings, and masks. Due to their high surface area and affinity for moisture, woven fabrics are prime habitats for microorganisms. At a minimum, these microbes deteriorate fabric structure and produce odors, but they can also harbor nosocomial bacteria and viruses that impact human health. Even though they are hydrophobic, nonwoven fabrics can also shelter these same microbes. For instance, a recent study demonstrated that the viability of SARS-CoV-2 virions was up to seven days on surgical masks [3]. For woven fabric, their pathogenesis is generally controlled by repeated laundering; but in nonwovens, microbes are typically eliminated by disposal or incineration [4].

Human respiratory pathogens such as bacteria, fungi, and viruses are increasingly responsible for significant morbidity and mortality in our modern society. According to the CDC, the 2019–2020 influenza season infected about 35 million people in the USA with 380,000 hospitalizations and 20,000 deaths [5]. However, this is insignificant compared to the COVID-19 pandemic, where SARS-CoV-2 variants have infected about 440 million people worldwide and caused about 6 million deaths as of March 2, 2022 [6]. Since airborne particles and aerosols are primary transmission routes for these microbes, facial coverings are critically important for source control. However, most masks only function as simple filtration devices [7–10]. Virus particles trapped in the mask can not only contaminate the wearer during daily use, but also be re-aerosolized during mask adjustments or removal [11]; and soiled masks represent a significant disposal biohazard [12]. This is unfortunate because virus viability on surgical masks and respirators is preventable. For instance, copper has been used in hospitals and common household items for centuries because of its antimicrobial characteristics. More recently, it has been incorporated into surgical masks [13, 14]. Several other antiviral agents have also been proposed for use in masks including polymeric biocides, nanoparticles of silver and zinc, iodine, chitosan, peptides, quaternary ammonium salts, polysaccharides, citrates, sodium-chloride, zeolites, graphene, graphene-oxide, and quantum dots [15–17]. The effectiveness of most of these compounds has yet to be clinically demonstrated; and their value remains debatable because they can be toxins, allergens, or irritants, limited in their antimicrobial efficacy, or environmental disposal hazards [13–15, 18–21].

Silicon nitride (Si_3_N_4_) is an alternative to these compounds. It is a US FDA cleared implantable biomaterial that has already passed a rigorous series of ISO-10993 human biocompatibility tests [22]. It has proven to be effective against a range of gram-positive and -negative bacteria [23–34] along with some fungi [35, 36], and its effectivity appears to be at least equivalent to other antimicrobial agents. Recent publications demonstrated its ability to rapidly inactivate viruses including SARS-CoV-2 [37–40]. In this study, it was hypothesized that non-woven fabric embedded with Si_3_N_4_ would not only trap viral droplets and particles, but also render them harmless. Incorporating this “catch and kill” mechanism into masks and their use by both the healthcare community and the general population could provide enhanced protection against the spread of respiratory disease. Therefore, the purpose of this study was to develop methods for embedding Si_3_N_4_ particles into hydrophobic polypropylene (PP) nonwoven fabric as an incorporated layer within a protective breathable mask, and subsequently test the efficacy of this fabric in inactivating SARS-CoV-2. This study was conducted in four phases: (1) The antiviral effectiveness of Si_3_N_4_ powder was first tested against a surrogate virus of lower pathogenicity (*i.e.*, human betacoronavirus, β-CoV, OC43); (2) Antiviral tests using Si_3_N_4_ powder were then performed against the alpha variant of SARS-CoV-2. Concurrent testing was also conducted using solid Si_3_N_4_ discs; (3) Si_3_N_4_-embedded nonwoven fabrics were then prepared and assessed for their antiviral effectiveness; and (4) Prototype masks or representative swatches were subjected to standard industrial tests for filtration efficiency, particle permeability and shedding, breathability, and chemical release. The results of this study demonstrated that Si_3_N_4_ powder, solids, and embedded fabrics were effective in reducing live SARS-CoV-2 virions by ~ 90% to 99.99% depending on Si_3_N_4_ type, concentration, and incubation time; and the other standard tests showed that Si_3_N_4_ prototype masks performed within permissible safety limits.

## Materials and Methods

### Test Materials

Test materials utilized in the study consisted of two Si_3_N_4_ powders (designated AP^2^ and AP^4^), sintered Si_3_N_4_ discs, and PP nonwoven fabric embedded with the two Si_3_N_4_ powders. Virogenic solutions (*i.e*., media) without Si_3_N_4_ powder, non-embedded PP fabric, and polyetheretherketone (PEEK) discs were used as controls.

**Fig. 1:**
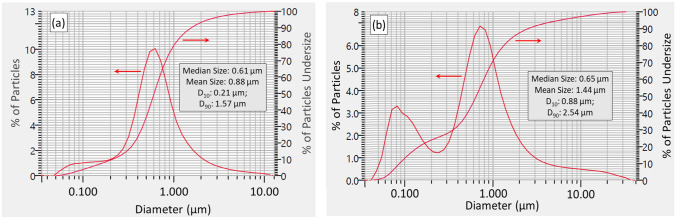
Particle size distributions for: **a** AP^2^ and **b** AP^4^ Si_3_N_4_ powders

The composition of the AP^2^ powder was nominally 90 wt% Si_3_N_4_ (Ube SN-E10, Ube Industries, Ube, Japan), 6 wt% yttrium oxide (Y_2_O_3_, Grade C, H.C. Starck, Goslar, Germany) and 4 wt% aluminum oxide (Al_2_O_3_, XRC-UFX, Baikowski International Corp, Charlotte, NC, USA). Preparation of this powder involved mixing and spray-drying of these raw materials, followed by a sequential series of firing operations including binder removal (~ 500 °C, 2 h, air), then densification in separate pre-sinter, sinter, and hot-isostatic pressing operations at temperatures between 1400 °C and 1750 °C for times of up to 3 h and N_2_ pressures of between 7 kPa and 200 MPa [41]. Between each of the firing steps, the powder was manually deagglomerated or crushed. The resulting grain was aqueously comminuted within an attrition mill for ~ 50 h, and the slurry was freeze-dried for ~ 4 days. The particle size distribution is shown in Fig. 1a. The AP^4^ powder was prepared without sintering additives; it was only subjected to air-firing (~ 300 °C, 1 h). Its particle size distribution is shown in Fig. 1b.

The Si_3_N_4_ discs (Ø12.7 × 1 ~ 2 mm) were produced using the same raw material composition as AP^2^ powder. They were machined from green-pressed blanks, pre-sintered, sintered, and hot-isostatically pressed [41]. After firing they were CO_2_ blasted, ultrasonically cleaned, and re-fired (700 °C, 2 h, air). Spunbond and melt-blown PP fabric was provided by O2TODAY™, (https://o2today.com/, Salt Lake City, UT, USA). They had nominal weights of 45 and 50 g/m^2^, respectively. The PEEK discs (Ø12.7 mm × 1 mm) were machined from rod stock provided by McMaster-Carr (ASTM D6262, https://www.mcmaster.com/, Aurora, OH, USA).

### Antiviral Testing of Si_3_N_4_ Powders

Antiviral assays using AP^2^ Si_3_N_4_ powder were performed against two human coronaviruses—the minimally pathogenic β-CoV, OC43 (obtained from ATCC) and the highly pathogenic SARS-CoV-2 (alpha variant, lineage A; isolate Hong Kong/VM20001061/2020, obtained from BEI Resources). For the OC43 tests, the Si_3_N_4_ powder was measured into Eppendorf tubes so that at 1 mL it would be either 7.5 or 15 wt%/vol. The OC43 virus was pre-prepared in a virogenic solution at a final TCID_50_ concentration of 7.34 × 10^7^/mL. The Eppendorf tubes were placed in an end-over-end tube rotator for specified periods (i.e., 1, 5, or 30 min). After incubation, supernatants were extracted and passed through a 0.45 µm filter, and TCID_50_ assays were conducted in accordance with procedures by Smither et al.[42]. Vero E6 mammalian kidney cells (ATCC CRL-1586™) were used as the infective host. This same procedure, but without addition of the virus, was employed for viability testing of Vero E6 cells in the presence of Si_3_N_4_. For the SARS-CoV-2 studies, the same procedure as described above was employed except that the initial TCID_50_ concentration was set to 3.16 × 10^6^/mL.

### Antiviral Testing of Solid Si_3_N_4_ Discs

Testing of solid disc surfaces was performed as specified in ISO 21702. As indicated previously, the test and control materials were Ø12.7 mm as-fired Si_3_N_4_ and PEEK discs, respectively. The discs were cleaned, disinfected, and sterilized by wiping with 70% ethanol. Both test and control specimens were analyzed for infectious virus titers immediately after inoculation, and after contacting the test discs for the specified time points (5, 10, and 30 min, and 24 h) at room temperature. Triplicate samples were used for all measurements. Virus-containing supernatants, at a concentration of 3 × 10^5^ TCID_50_/ml, were applied to each disk. At the specified time points, media was removed to a new tube and a series of 4–1 mL washes was performed. All the media was mixed into the tube and the viral titer was determined by TCID_50_ assays.

### Antiviral Testing of Nonwoven Fabric Embedded with Si_3_N_4_ Powder

**Fig. 2:**
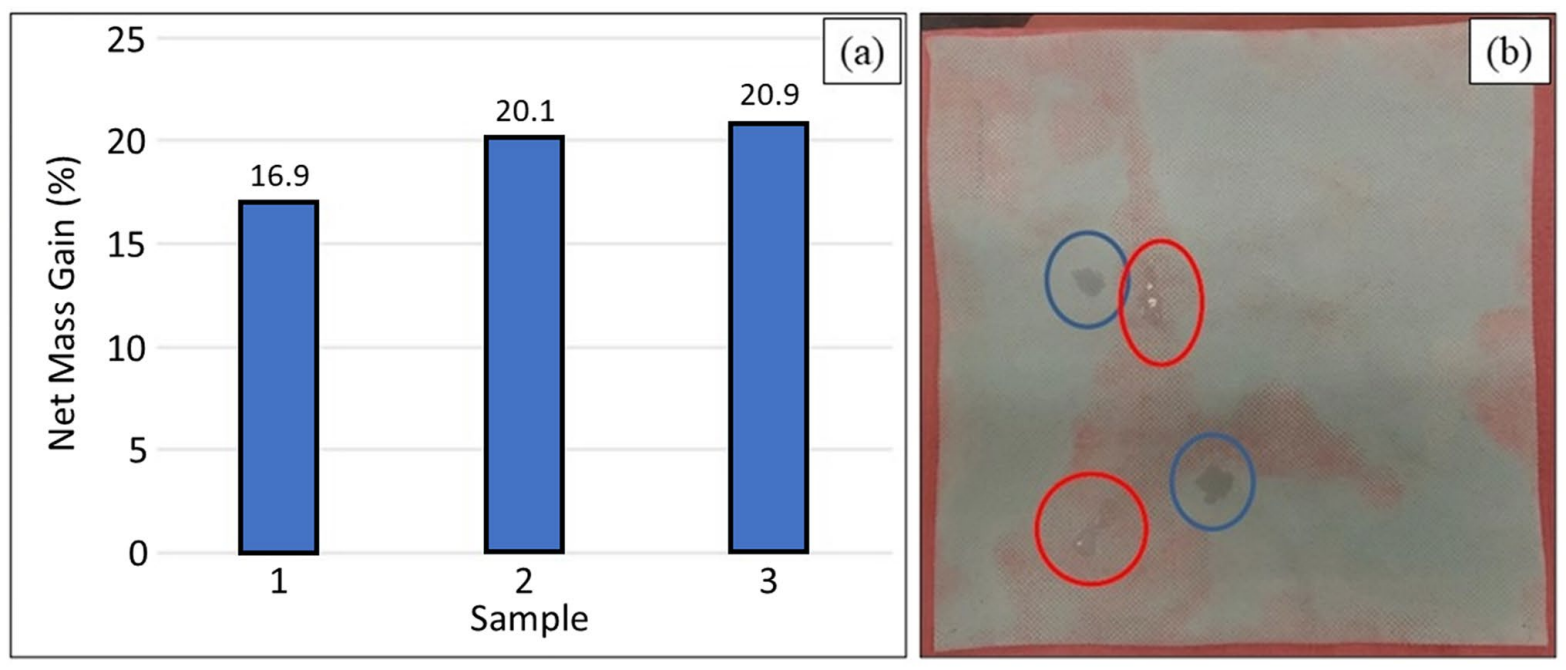
**a** AP^2^ Si_3_N_4_ mass gain for three fabric samples; and **b** Hydrophilic characteristics of sample 1. Blue circles indicate reasonable wetting behavior. Red circles are non-wetting. The non-wetting areas corresponded to poor Si_3_N_4_ powder infiltration

**Fig. 3:**
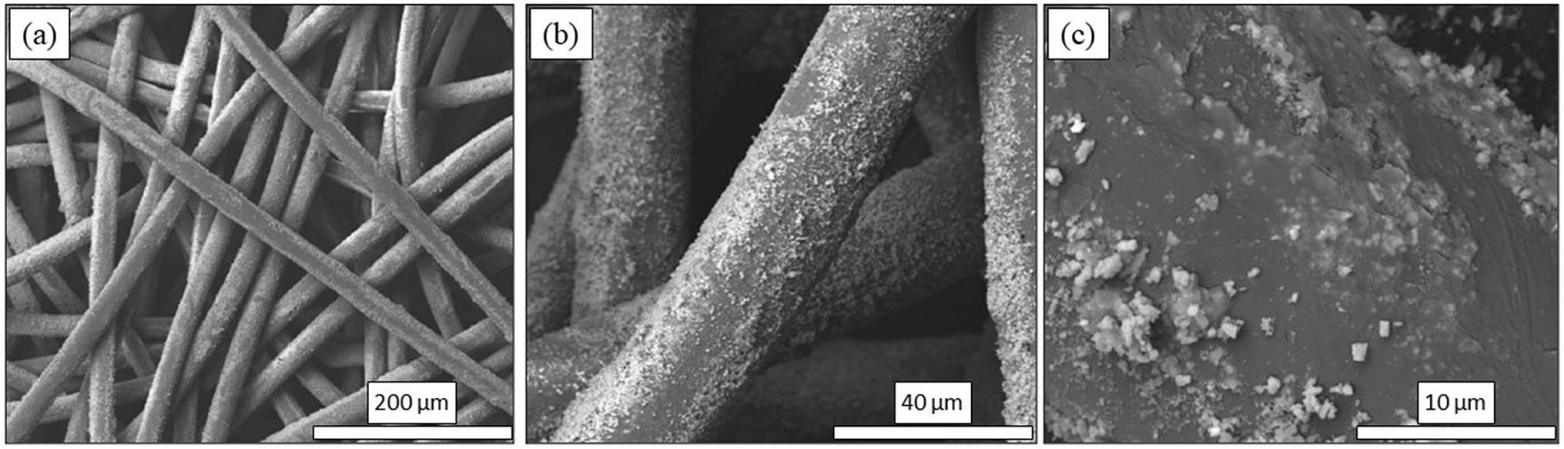
SEM photomicrographs of spunbond PP fibers coated with AP^2^ Si_3_N_4_ particles: **a** ×500, **b** ×2500, and **c** ×10,000 magnification

Preparation of the nonwoven Si_3_N_4_-embedded fabric was based on the “pad-dry-cure” method [43]. Both AP^2^ and AP^4^ Si_3_N_4_ powders were utilized. Pre-treatment of the PP fabric was necessary due to the fibers’ highly hydrophobic nature. For the AP^2^ powder, this involved pre-rinsing the fabric under mechanical agitation in deionized (DI) H_2_O (100 °C, 5 min) followed by addition and adsorption of a surfactant (0.6 wt% dodecyl trimethyl-ammonium bromide, DTAB, Sigma Aldrich, CAS 1119-94-4) for 30 ≤ t min ≤ 90. The fabric was oven dried (110 °C, 10 min, air) and spray-coated with ~ 5 vol% aqueous slurry of AP^2^ Si_3_N_4_, followed by immersion and sonication in the AP^2^ slurry (10 min, 60 °C). The fabric was wrung of excess slurry, oven dried (20 min, 110 °C), and cured between heated weighted plates (145 °C, 90 min, 1.4 kPa). To remove non-adherent Si_3_N_4_ particles, the fabric was washed using 1 vol% Triton X-100 (Sigma Aldrich, CAS 9002-93-1) under sonication (65 °C, 5 min) and rinsed in clean DI H_2_O five times followed by ~ 30 s compressed air blow-out (690 kPa). To increase powder loading, this entire procedure following fabric pre-treatment with DTAB was repeated. The post-processing net mass gain of three sampled swatches, shown in Fig. 2a, averaged 19.3 wt%. Figure 2b provides a view of one of the swatches. Note that embedding of the Si_3_N_4_ was non-uniform. Although most sections of the fabric were adequately covered with powder, minor sections had less than optimal Si_3_N_4_ content. A qualitative water-drop hydrophilicity test was performed on these sections. It was noted that areas of heavy powder concentration exhibited improved hydrophilicity (*cf.*, Fig. 2b). Scanning electron microscopy (SEM, FEI Quanta 600 FEG, 10 kV) images were acquired on representative sections of the fabric. The powder was found to be reasonably dispersed and partially embedded into individual fibers as shown in Fig. 3a–c.

**Fig. 4:**
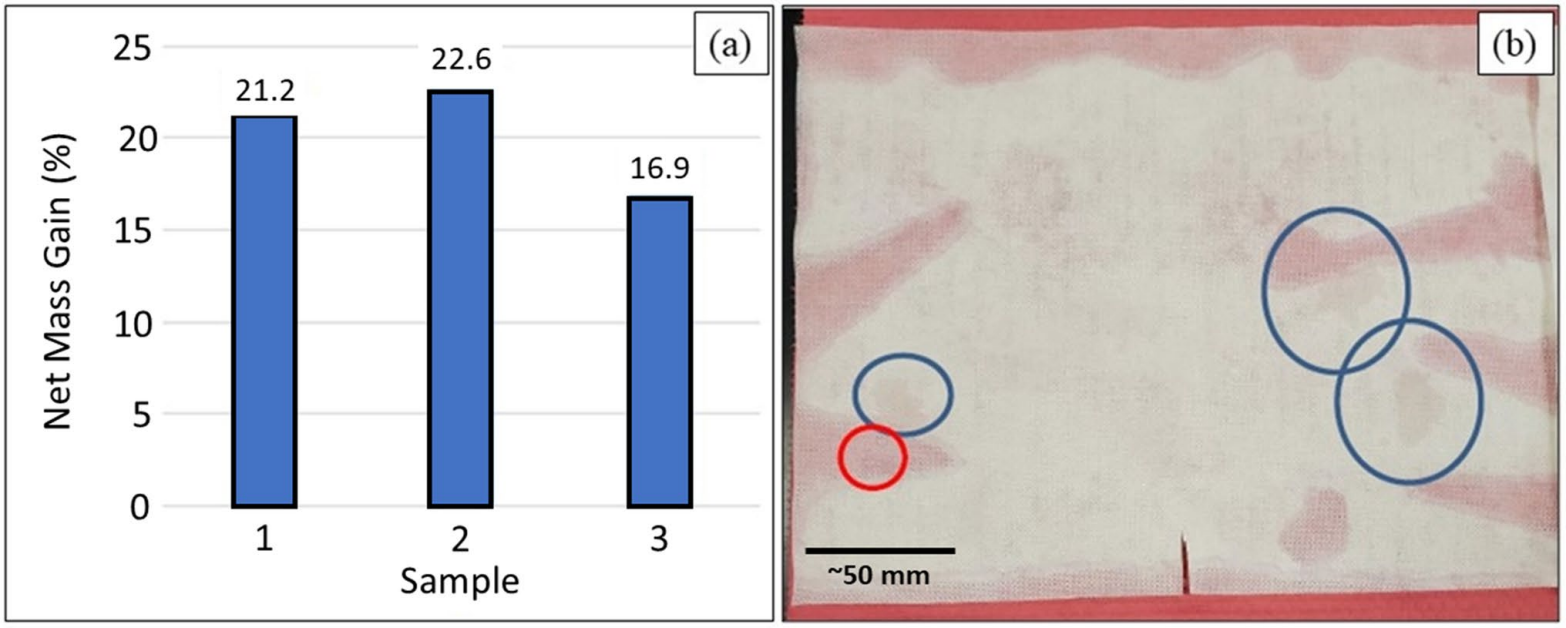
**a** AP^4^ Si_3_N_4_ mass gain for three fabric samples; and **b** Hydrophilic characteristics of sample 1. Blue circles indicate reasonable wetting behavior. The red circle is non-wetting. The non-wetting areas corresponded to poor Si_3_N_4_ powder infiltration

**Fig. 5:**
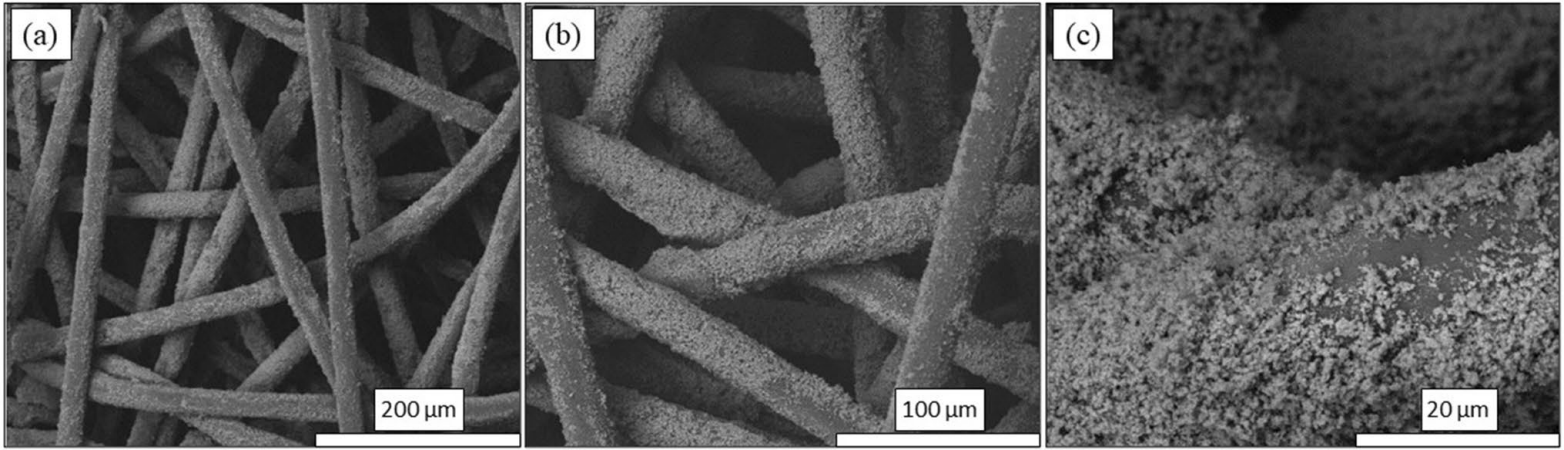
SEM photomicrographs of spunbond PP fibers coated with AP^4^ Si_3_N_4_ particles: **a** ×500, **b** ×1000, and **c** ×5000 magnification

A similar procedure was utilized for embedding the AP^4^ powder. Pretreatment occurred by sonication in DI water (95 °C, 10 min), followed by DTAB absorption (100 °C, 30 ≤ t min ≤ 120) and oven drying (110 °C, 10 min). The fabric was then immersed in an aqueous AP^4^ slurry (8 vol%, 10 min, 60°C), wrung of excess slurry, oven dried (110°C, 10 min), and cured between heated weighted plates (145 °C, 90 min, 1.4 kPa). Washing and rinsing included sonication in DI water with 1 vol% Triton X-100 (60 °C, 5 min), followed by a sonicated DI water rinse (50°C, 5 min), oven drying (110°C, 10 min), and ~30 s compressed air blow-out (~ 690 kPa). The post-processing net mass gain for three representative samples, shown in Fig. 4a, averaged 20.2 wt%. Figure 4b provides a view of one of the swatches. SEM results for the AP^4^-embedded spunbond PP layer are presented in Fig. 5a–c. Results for the AP^4^ fibers were similar to the AP^2^ fabric for embedded mass, wetting, and non-wetting areas, dispersion, and adherence.

Antiviral test swatches of both the AP^2^ and AP^4^ fabric were prepared by cutting square (20 or 50 mm) sections, and selecting only areas that were evenly embedded with the Si_3_N_4_ powder. Multiple fabric sections were then assembled using ultrasonic welding to form individual multilayer swatches weighing about 0.36 ± 0.01 g in accordance with the sample mass of 0.40 ± 0.05 g specified by ISO 18184. All fabric specimens were then autoclaved inside 30 mL screwcap, polypropylene vial containers prior to antiviral experimentation. The treated test specimens (virus-containing supernatants) were analyzed for infectious virus titers immediately after inoculation, and after contacting the test specimens for the specified time points (5, 10, 30, 120 min) in accordance with ISO 18184. Triplicate samples were used for all measurements.

### Mask Tests

Four- or five-layer prototype masks and representative mask swatches were prepared for standard industrial mask tests including ASTM F2299 and EN 13274-7 for filtration efficiency, EN 14683 for differential pressure drop, and ISO 18562-2 for particle shedding. The prototype masks and swatches consisted of outer spunbond PP fabric layers (45 g/m^2^), inner melt-blown layers (~ 50 g/m^2^), and a central spunbond layer embedded with AP^4^ Si_3_N_4_ powder (20 to 30 wt%) sandwiched between the other layers. The fabric layers were ultrasonically welded together. Testing was conducted by two certified laboratories—Nelson Laboratories (Salt Lake City, UT, USA, ASTM F2299, ISO 18562-2) and Intertek Testing Services, Ltd (Shanghai, China, EN 13274-7, EN 14683). In addition, a modification of ISO 18562-3 was developed to detect ammonia (NH_3_) release from the Si_3_N_4_ powders. This protocol involved equilibrating 1 g of AP^2^ or AP^4^ powder within a closed 500 mL clamshell reactor for 30 min, then subsequently measuring NH_3_ concentrations within the chamber under either static (30 min) or flowing air (~ 28.3 L/min, 5 min) at ambient (22 °C, 35% RH) or elevated (60 ~ 100 °C, 99.9% RH) conditions. Released NH_3_ was measured using industrial 5–100 ppm colorimetric gas detection tubes (www.sensidyne.com, St. Petersburg, FL, USA).

### Statistical Analysis

A linear mixed model was used to study the change of viral titers over time for each material. The significance level was set at 0.05 for each comparison. The analysis was implemented with SAS 9.4 software (SAS Institute Inc., Cary, NC, USA).

## Results

### In Vitro Antiviral Testing of Si_3_N_4_ Powders

**Fig. 6:**
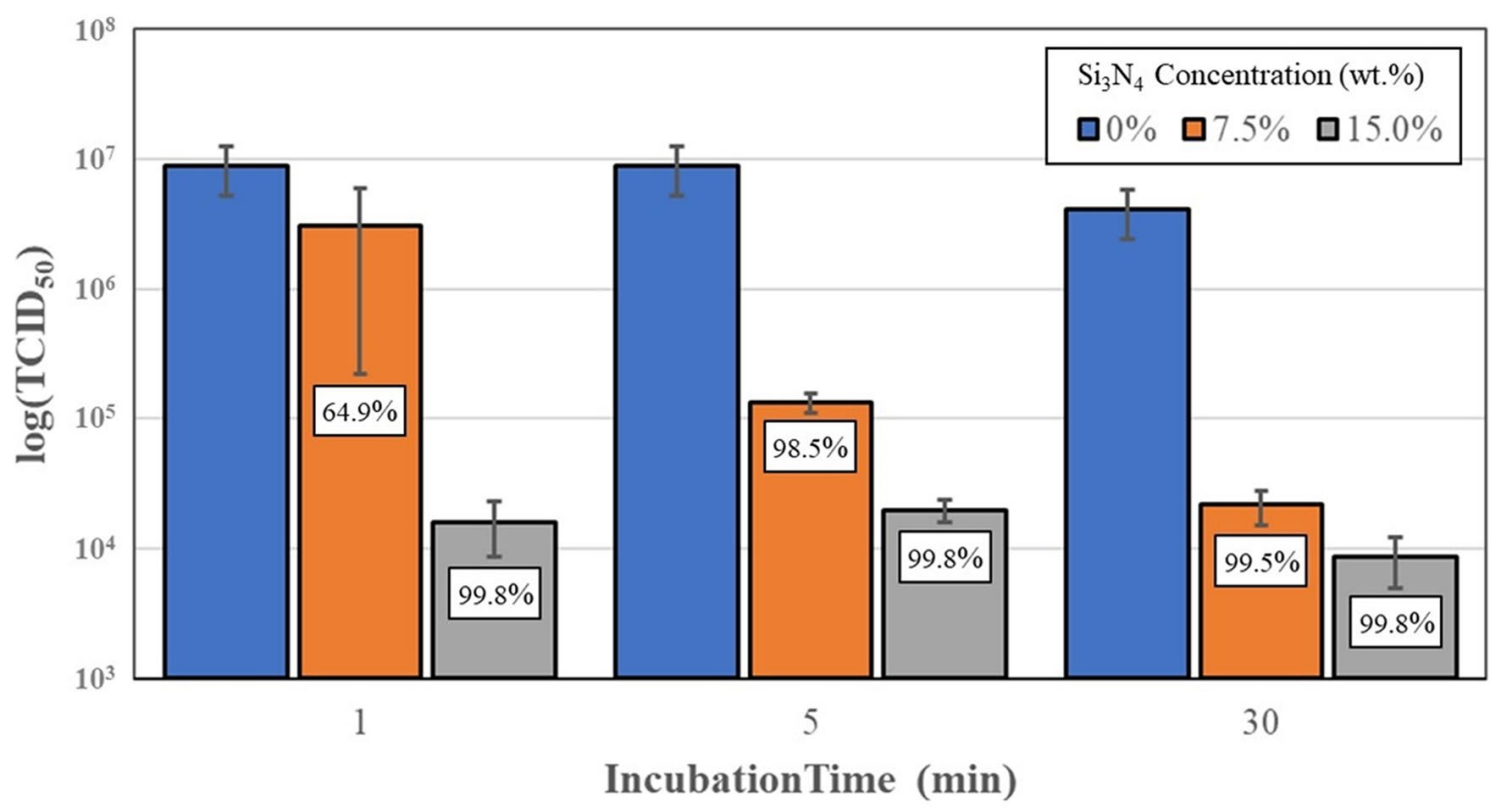
Virus titers and % OC43 β-CoV inactivation, after incubation with 7.5 and 15.0 wt%/vol. AP^2^ Si_3_N_4_ powder. Error bars represent the standard error of the means

The first series of tests used the OC43 human β-CoV as a surrogate for SARS-CoV-2. The pathogenicity of the β-CoV is significantly lower than SARS-CoV-2 and therefore it could be reasonably handled in a BSL-2 laboratory. The results of these tests are graphically presented in Fig. 6. The OC43 β-CoV was essentially inactivated on contact with AP^2^ Si_3_N_4_ powder. Reductions of 64.9% and 99.8% occurred within one minute at concentrations of 7.5 and 15.0 wt%/vol. of Si_3_N_4_, (*p* = 0.29 and 0.07), respectively. Within five minutes, viral reductions were 98.5% and 99.8% (*p* = 0.08 and 0.07), respectively; and after thirty minutes, viral loads were reduced by 99.5% and 99.8% for the 7.5 and 15.0 wt%/vol. concentrations (*p* = 0.07 and 0.07), respectively. Vero E6 cell viability testing was conducted by exposing the mammalian cells to the Si_3_N_4_ powder in the virogenic medium, but without adding the virus. No cell death was observed at either Si_3_N_4_ concentration or at any of the incubation time points (data not shown). Mammalian cell viability was therefore deemed to be 100% in the presence of the dispersed Si_3_N_4_ powder.

**Fig. 7:**
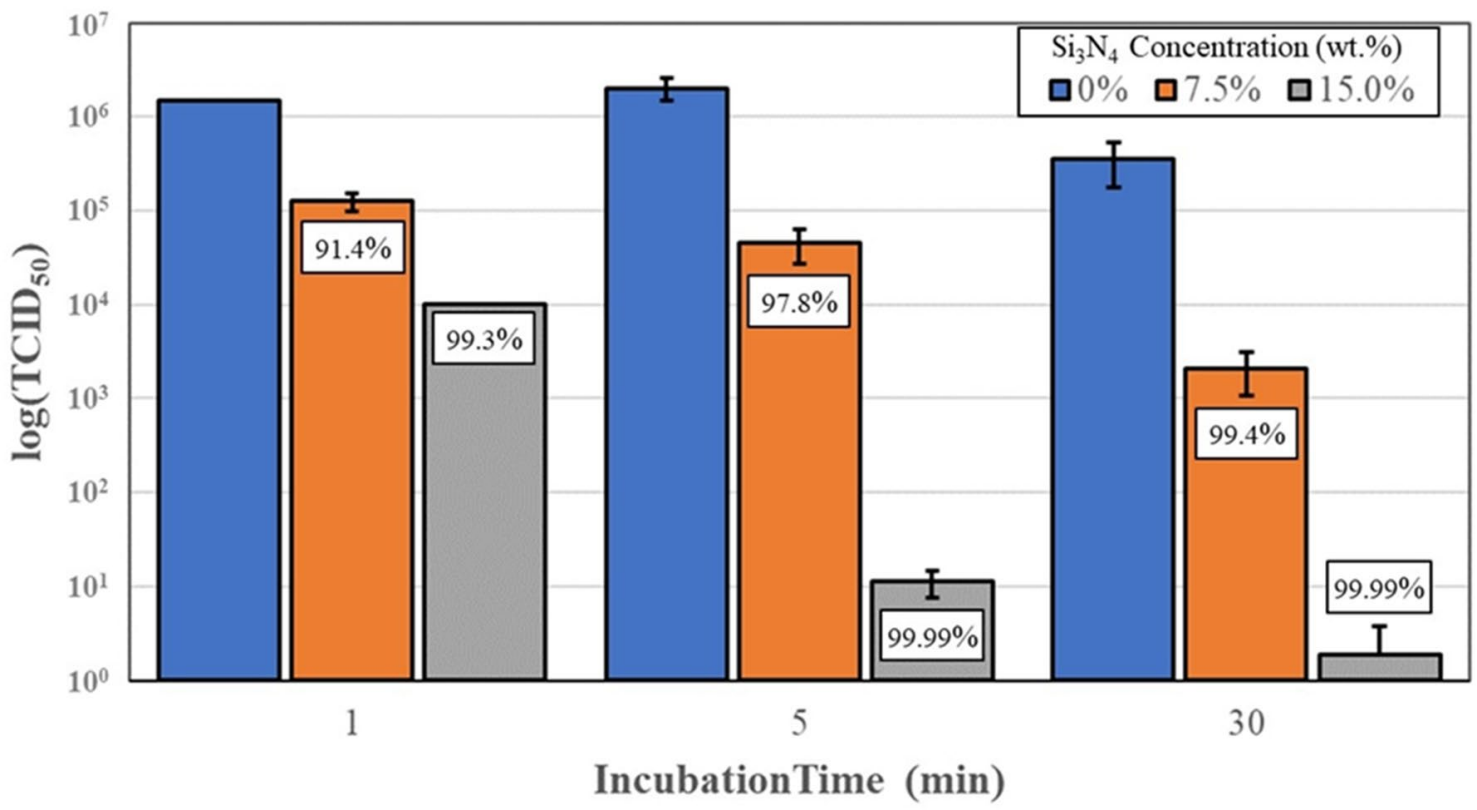
Virus titers and % SARS-CoV-2 virus inactivation, after incubation with 7.5 and 15.0 wt%/vol. AP^2^ Si_3_N_4_ powder. Error bars represent the standard error of the means

After completing tests using the β-CoV, activities were moved into the BSL-3 laboratory for testing with SARS-CoV-2. Results of these experiments are provided in Fig. 7. At one minute of exposure to 7.5 and 15 wt%/vol Si_3_N_4_ powder, the viral load was reduced by 91.4% and 99.3% (*p* =  < 0.01 and < 0.01), respectively. A five-minute exposure resulted in reductions of 97.8% and 99.99% for the two powder concentrations (*p* = 0.02 and 0.02), respectively; and at thirty minutes of exposure, reductions were 99.4% and 99.99% for 7.5 and 15 wt%/vol., (*p* = 0.12 and 0.12), respectively.

### In Vitro Antiviral Testing of Si_3_N_4_ Solids

**Fig. 8:**
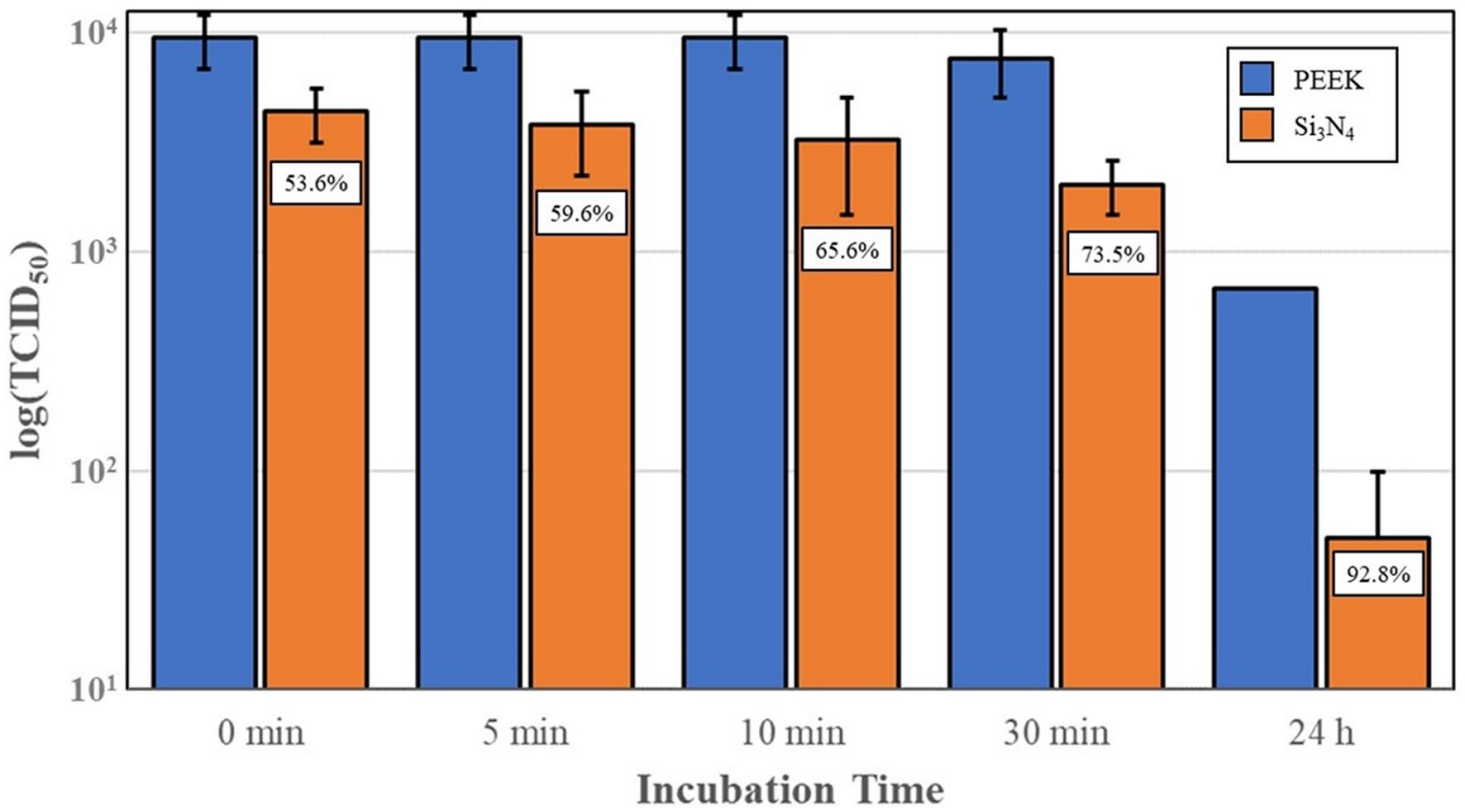
Virus titers and % SARS-CoV-2 virus inactivation, after incubation with PEEK and *MC*^*2*^ Si_3_N_4_ solid discs. Error bars represent the standard error of the means

As an additional analysis of the efficacy of Si_3_N_4_ as an antiviral compound, SARS-CoV-2 antiviral assessments were also conducted using solid Si_3_N_4_ and PEEK discs. Results of these tests are presented in Fig. 8. The data show that SARS-CoV-2 was strongly inactivated upon contact with solid Si_3_N_4_, but virus inactivation on the solid discs was lower than that of the powders. Inactivation totals were 53.6%, 59.6%, 65.6%, 73.5% and 92.8% at 0, 5, 10, and 30 min and 24 h, (*p* = 0.16, 0.14, 0.15, 0.11, and < 0.01), respectively. As noted, significant virus incubation time was required to achieve greater than a 2-log reduction. This was likely due to the reduced surface area of the discs in contact with the virogenic medium.

### In Vitro Antiviral Testing of Nonwoven Fabric Containing Si_3_N_4_ Powder

**Fig. 9:**
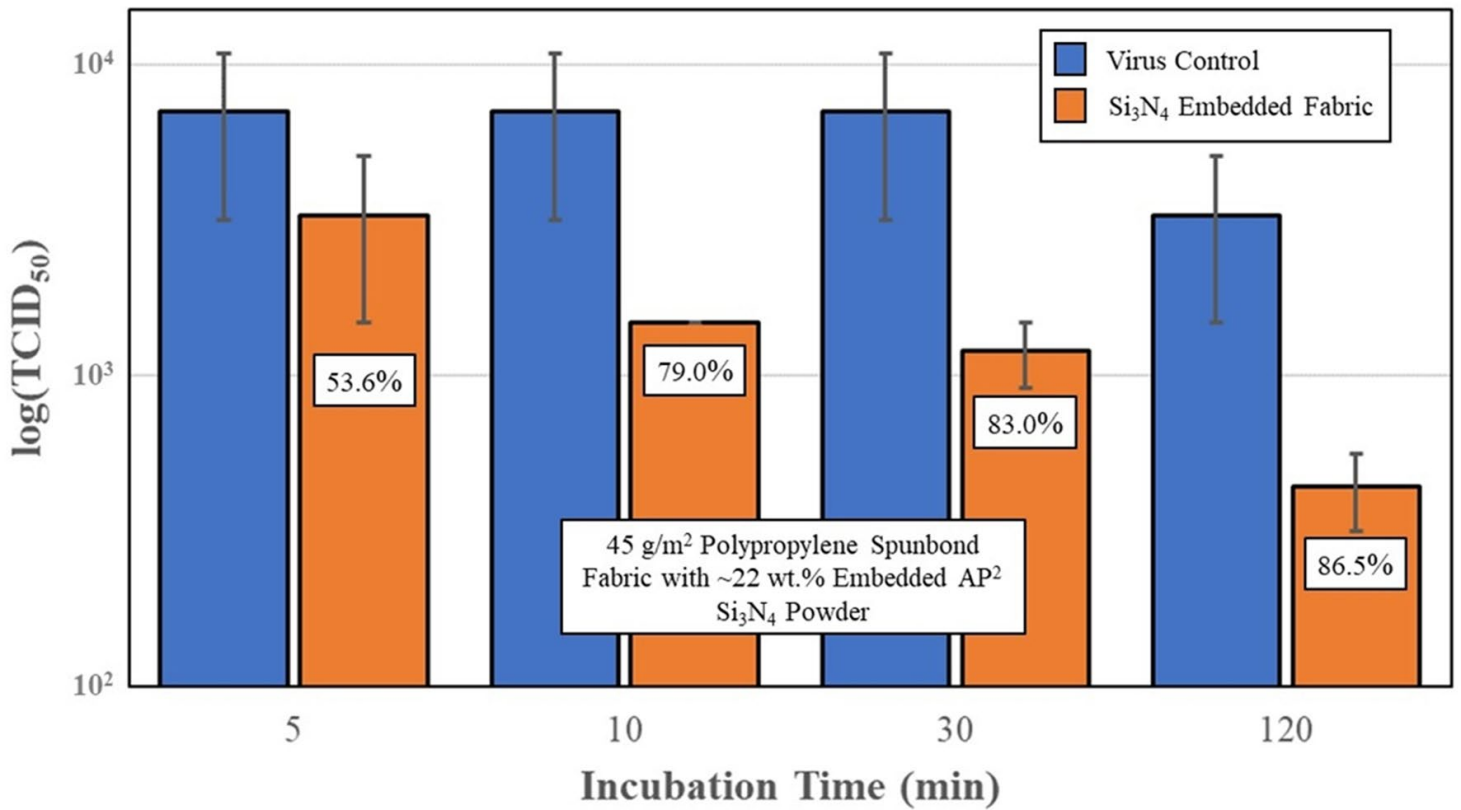
Virus titers and % SARS-CoV-2 virus inactivation, after incubation with polypropylene fabric embedded with AP^2^ Si_3_N_4_ powder. Error bars represent the standard error of the means

**Fig. 10:**
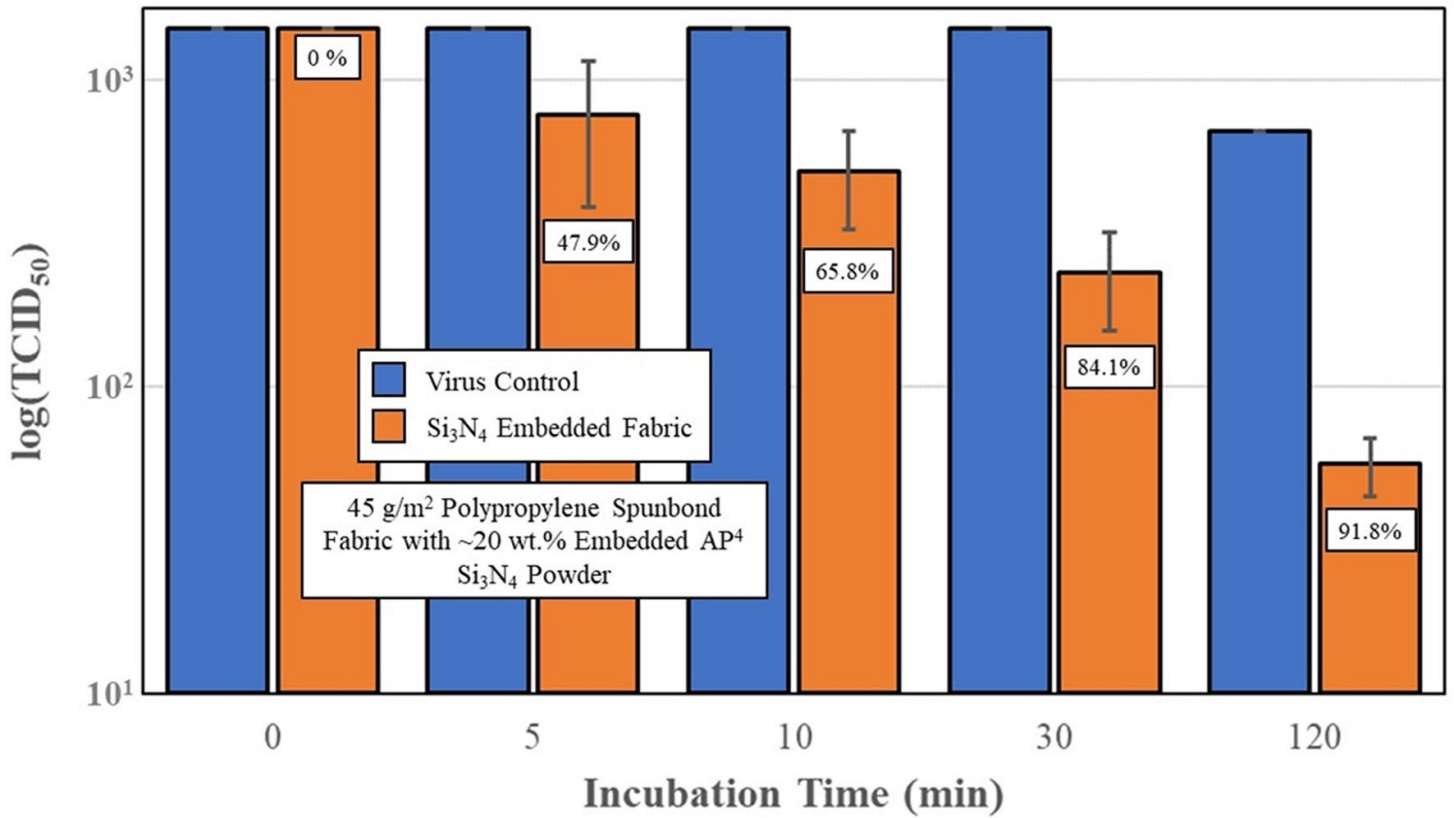
Virus titers and % SARS-CoV-2 virus inactivation, after incubation with polypropylene fabric embedded with AP^4^ Si_3_N_4_ powder. Error bars on the embedded fabric represent the standard error of the means. The standard errors of the means for the virus controls were zero

Lastly, Si_3_N_4_-embedded nonwoven fabric was prepared and assessed for its SARS-CoV-2 antiviral effectiveness. Two series of tests were conducted using AP^2^ and AP^4^ powder. Results are presented in Figs. 9 and 10, respectively. The AP^2^ Si_3_N_4_-embedded fabric showed approximately 50% reduction in viral load after 5 min of incubation. Progressive inactivation of the virus at longer time points occurred with viral load reductions of 79%, 83% and ~ 87% at 10, 30, and 120 min, (*p* = 0.43, 0.22, 0.21, and 0.19), respectively. Similar reductions were also noted for the AP^4^-embedded fabric, with inactivation rates of approximately 48%, 66%, 84%, and 92% at 5, 10, 30, and 120 min (*p* = 0.14, < 0.01, < 0.01, and < 0.01), respectively. Although a greater than 2-log reduction was observed at 120 min, the antiviral efficacy of the fabric samples was also lower compared to the powders. This observation is presumed to be due to the hydrophobic nature of the spunbond PP fibers which likely inhibited intimate contact between the embedded powder particles and the virogenic solution.

### Mask Tests

**Fig. 11:**
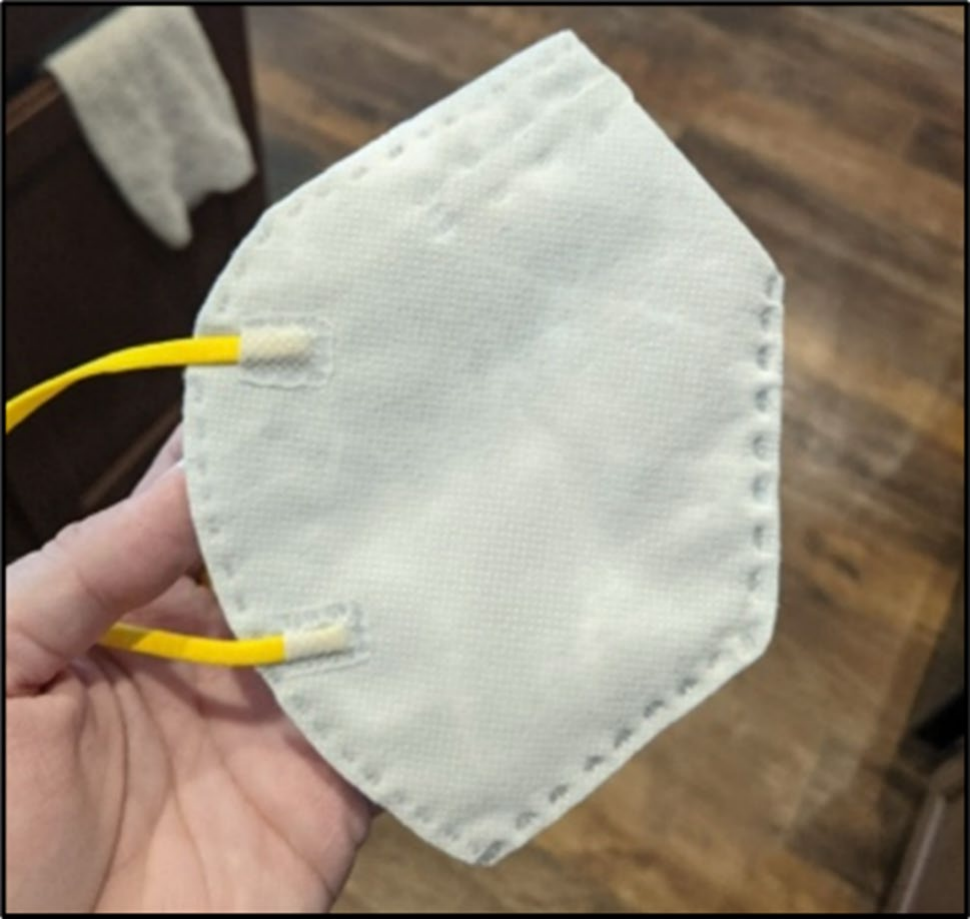
Prototype Si_3_N_4_ Mask

An example of a prototype mask is shown in Fig. 11, and the results of the various standard industry tests are provided in Table 1. Independent laboratories assessed filtration efficiency using two methods: the salt aerosol technique (EN 13274-7) and the latex particle challenge (ASTM F2399). Both tests gave similar results—99.18% and 99.97%, respectively, indicating that the test masks exceed the N95 particle filtration standard. ISO 18562–2 was used to assess the risk of Si_3_N_4_ particle release from the mask fabric. Average values for PM_2.5_ and PM_10_ were identical at 1.25 ± 0.5 µg/m^3^, and both were well within permissible limits of 12 and 150 µg/m^3^, respectively. Breathability of the test masks was determined using EN 14683 which measures differential pressure though the fabric. The resulting drop in pressure was 53 ± 2 Pa/cm^2^. This value meets EU and US specifications for Type IIR medical masks for medium splash protection (i.e., < 60 Pa/cm^2^), but the outcome was slightly higher than the Type I requirement (< 40 Pa/cm^2^).

**Table 1:** Results from standard industrial mask tests

Test standard	Description	Methodology	n	Test result	
EN 13274-7	Particle filtration efficiency (PFE)	Sodium chloride Aerosol penetration	5	% Filtration efficiency	99.18 ± 0.37
ASTM F2299	Particle filtration efficiency (PFE)	Latex particle challenge	4	% Filtration efficiency	99.97
ISO 18562-2	Particulate matter emissions	PM_2.5_ and PM_10_ for 24 h in 3.0 LPM Air	4	PM_2.5_ (µg/m^3^)	1.25 ± 0.5
				PM_10_ (µg/m^3^)	1.25 ± 0.5
EN 14683	Differential pressure test	Breathing resistance	5	∆ Pressure (Pa/cm^2^)	53 ± 2

Because the antipathogenic mechanism of Si_3_N_4_ is due to a hydrolytic surface reaction that converts the nitride to an oxide with the release of nitrogen [32], a special test was developed based on ISO 18562-3 to detect the presence of ammonia gas. Instead of testing with fabric directly, this test was conducted using both the AP^2^ and AP^4^ Si_3_N_4_ powders at masses that were at least eight times the equivalent amount of powder embedded into an individual mask. Results for this test are provided in Table 2. Of the three test environments, static air conditions resulted in 80 ppm NH_3_ (equivalent to 10 ppm or less for one mask-equivalent powder load) for the AP^2^ Si_3_N_4_, but none in flowing air. The AP^4^ powder showed no detectable NH_3_ regardless of test condition. Various regulatory agencies have established limits on time-averaged occupational exposure to NH_3_. The American Conference of Governmental Industrial Hygienists (ACGIH) set an 8 h time-weighted exposure of 25 ppm and a 15 min short-term exposure (STEL) of 35 ppm. US OSHA has an 8 h permissible limit of 50 ppm, whereas NIOSH and California OSHA recommend a 10 h limit of 25 ppm and a 15 min STEL of 35 ppm (https://www.cdc.gov/niosh/pel88/7664-41.html). Although the methods for performing workplace tests differ from this mask test, it is clear that AP^4^ Si_3_N_4_ powder is within permissible exposure levels whereas the AP^2^ powder remains questionable. The reason the AP^2^ powder has higher NH_3_ emissions is likely due to the formation of an amorphous silicon-yttrium–aluminum oxynitride (SiYAlON) phase during the sintering and hot-isostatic pressing operations (*c.f.*, Section “Test Materials”), but further confirmatory research is underway.

**Table 2:** Results from modified ISO 18562–3 Standard for NH_3_ detection

Test conditions	Si_3_N_4_ test material	
	AP^2^	AP^4^
Static ambient Air, 30 min sampling (ppm)	80	N.D
Static 99.9% RH Air, 30 min sampling (ppm)	80	N.D
Dynamic ambient air, 28.3 L/min, 5 min sampling (ppm)	N.D	N.D

*N.D.* not detectable

## Discussion

Respiratory transmission of the SARS-CoV-2 virus has accentuated demand for masks. As a result, hundreds of merchants have responded and now offer facial coverings, many including antimicrobial agents. However, few have the technical expertise to supply personal protective equipment (PPE); and while a limited number have performed third party antipathogenic testing, even fewer have conducted studies against SARS-CoV-2. Those that are reporting effectivity against the COVID-19 virus typically use metal or metal oxide nanoparticles. Most companies are startups with little or no experience in large-scale manufacturing and FDA or EPA regulatory requirements [17]. As examples, Jung et al*.* prepared a highly breathable mask that was vacuum coated with Cu and subsequently oxidized to CuO. They observed a 75% reduction in viral load after 1 h incubation with SARS-CoV-2 [44]. Borkow reported a similar result for Cu coated masks with 99.9% inactivation of the pathogen within 1 min using TCID_50_ and PCR assays [45]. Balagna et al*.* developed a silver cluster/silica composite sputter coating onto a mask and observed a 2- to 4-log reduction in SARS-CoV-2 after 1.5 h incubation [46]. Gopal utilized ZnO nanoparticles embedded in water-absorbable 6′6-nylon fibers to develop a mask that was 99% effective (i.e., 2-log reduction) against SARS-CoV-2 in less than 1 h using a modified ISO 18184 protocol [47]. Marti, et al*.* reported on the development of a nonwoven face mask embedded with benzalkonium chloride. They found it to be capable of inactivating more than 99% of SARS-CoV-2 in one minute. They also found it to be effective against methicillin-resistant *S. aureus* and *S. epidermidis*. However, they concluded that significant additional testing will be required to ensure the safety and correct usage of their technology for mass production and commercialization [48]. Their conclusion is valid for all the foregoing mask concepts.

The plethora of vendors who have jumped into the market has prompted the publication of several critical and systematic reviews on masks containing antimicrobial agents [49–53]. For instance, Stokes et al*.* employed PRISMA guidance [54] to select 17 articles from 2,116 records specifically addressing the use of antimicrobial agents in medical and community face masks [49]. Although their review focused mainly on methodologies for determining antimicrobial effectiveness, the cited articles revealed that all masks were in development or were laboratory prototypes. Antimicrobial agents included metal oxides and nanoparticles, N-halamines, quaternary ammonium compounds, salts, graphene, iodine, and naturally derived substances. None had received regulatory approval or were tested against SARS-CoV-2. In a separate review, Chua et al*.* evaluated 12 masks, of which six were either N95 respirators or surgical masks, while the remaining were consumer oriented. Embedded antimicrobial agents included citric acid, and/or nanoparticles of copper (Cu), copper iodide (CuI), zinc (Zn), silver (Ag) or their respective oxides (i.e., Cu_2_O, Ag_4_O_4_, and ZnO). None of these masks were evaluated for their effectiveness against SARS-CoV-2 either, but all broadly claimed to be capable of eliminating virus, bacteria, and fungi [50]. In a more recent review, Pullangott et al*.* identified 17 commercial antimicrobial masks by brand name. Agents in these masks included metal or metal-oxide nanoparticles (*e.g.*, Cu, Zn, Ag, or mixtures), iodine, salts, organosilanes, or graphene. Detailed investigation of product websites revealed that only four had been cleared by the FDA as either N95 respirators or surgical masks, twelve were consumer masks, and one was not a mask at all, but an antimicrobial face spray. Four of the masks claimed to be effective against SARS-CoV-2, but test results were not provided. Blevens et al. also conducted a more recent review, but focused solely on consumer cloth masks that contained silver as the antimicrobial agent [52]. They investigated claims for 40 masks by assessing patents, regulatory certifications, EPA registrations, and peer-reviewed publications. They concluded that 19 of the 40 had unsubstantiated claims (47%); and recommended stricter government regulations to ensure the efficacy of advertised products. A systematic review by Carvalho, et al. predominantly found that most contemporary research emphasizes use of silver, copper, and polymer-based nanomaterials as the primary agents against SARS-CoV-2 [53]. Yet, due to the disposable nature of masks and the longevity of the embedded compounds, they raised a valid environmental concern. Once these elements or compounds are released, they cannot be easily recovered, and will eventually pollute both animal and human food chains. Silver, copper, and zinc are known to be toxic above nutrient levels, and their unbridled use appears to be outpacing regulatory controls [55–60]. This concern was further emphasized in a separate critical review by Pollard, et al*.* They obtained samples of nine silver or copper impregnated masks and subjected them to a DI water soak, saliva tests, and up to ten simulated household laundry cycles [61]. They found a significant amount of the nanometal particles were leached into the graywater during washing – in some cases up to 100%. In fact, one mask lost 52% of its copper during the 1 h DI water rinse, and the remainder during its initial wash cycle. All masks showed sensitivity to saliva with one mask exhibiting 20% leaching of copper over an 8 h period. The authors expressed concern over the use of these masks, not only for the environmental effluent, but also for the toxicity they pose to the wearer.

It is important to note that the FDA defines products that are intended for the “diagnosis of disease or other conditions or in the cure, mitigation, treatment, or prevention of disease” as medical devices. This includes antimicrobial masks. The FDA published (2004) and has subsequently revised (2020–2021) guidance for respirators, surgical, and commercial masks [49–51]. All N95 respirators and surgical masks must receive NIOSH and/or FDA clearance regardless of the inclusion of antimicrobial agents. Companies marketing masks with embedded agents that inhibit respiratory diseases must also receive FDA clearance prior to market release. Or, they must not post any efficacy claims and provide a disclaimer that clearly states that their products have not been reviewed by the FDA, are not to be used in a medical setting, and are not intended to protect users from disease. But this hasn’t dissuaded numerous unscrupulous groups from marketing and selling masks claiming to be effective against airborne pathogens, while providing little or no supporting evidence, and no regulatory approvals. In response, the FDA is systematically reviewing websites and notifying violators.

In light of this information, the current study was undertaken as an initial foray into the development of a novel antipathogenic mask in advance of commercial considerations. The objective of the study was to perform a reasonable evaluation of a potential product in accordance with accepted industrial standards. The study methodically encompassed testing of powders, solids, and embedded nonwoven fabric against two human coronaviruses—the minimally pathogenic OC43 β-CoV (which was initially utilized as a surrogate) and the SARS-CoV-2 virus. Using all three forms of Si_3_N_4_, the results showed that this unique material was effective in reducing viral loads. The data confirm that powders produced the greatest viral reductions *(i.e*., up to 99.99% after 30 min of exposure, *cf*., Fig. 7), whereas solids and fabric were less effective (*i.e*., ~ 87% to 92%, *cf*., Figs. 8, 9, 10). The lower surface area of the solid Si_3_N_4_ discs is likely the reason for their reduced efficacy; and for the embedded fabric, it is postulated that the multilayer requirement of the ISO 18184 protocol coupled with the hydrophobic nature of the PP fabric likely limited intimate contact between the virogenic medium and the embedded Si_3_N_4_ particles. However, regardless of form, the results provide consistent evidence that Si_3_N_4_ is an effective antipathogenic agent against SARS-CoV-2. In addition, the study examined important mask safety features including filtration efficiency, differential pressure, particle shedding, and chemical release. These results indicated that prototype masks (or mask swatches) substantially met filtration, breathability and particle shedding standards. A modified chemical release protocol showed no detectable ammonia from at least one of the test Si_3_N_4_ powders. Lastly, although not evaluated in this study, Si_3_N_4_ is not expected to be an environmental hazard like most other antimicrobial agents. Si_3_N_4_ is composed of the two most abundant elements in the earth’s crust and atmosphere (*i.e*., silicon and nitrogen), respectively. In summary, although this study provides credible evidence of the efficacy of Si_3_N_4_-embedded fabric against a critical respiratory pathogen, the authors recognize that significant additional development, testing, and regulatory approvals will be necessary before effective PPE can be released to the medical community or the general population.

## Conclusions

Si_3_N_4_ powders, solids, and embedded PP fabrics were tested for their antiviral efficacy against SARS-CoV-2 with viral load reductions of 99.99% at ≤ 5 min (powders), ~ 93% in 24 h (solids), and 87% ~ 92% in 120 min (embedded fabrics), respectively. For all three Si_3_N_4_ materials, virus inactivation was found to be concentration and time dependent (*i.e.*, greater reductions in viral titers were observed at higher Si_3_N_4_ concentrations and longer exposure times). Prototype masks (or mask swatches) were also evaluated for filtration efficiency, differential pressure, particle shedding, and chemical release. Results of these standard mask tests were generally within prescribed safety limits. Given this initial study, Si_3_N_4_-embedded nonwoven fabric may represent an advancement in the fight against respiratory diseases. Its incorporation into facial masks may upgrade personal protective devices from simple “capture and retain” to effective “capture and kill” protection.

## Patents

The following US and International pending patents are a partial result of this study:

1. McEntire et al. [62]
2. McEntire et al. [63]
3. McEntire et al. [64]
4. McEntire et al. [65]
